# Ketogenic Diet Ameliorates Cardiac Dysfunction via Balancing Mitochondrial Dynamics and Inhibiting Apoptosis in Type 2 Diabetic Mice

**DOI:** 10.14336/AD.2019.0510

**Published:** 2020-03-09

**Authors:** Yongzheng Guo, Cheng Zhang, Fei-Fei Shang, Minghao Luo, Yuehua You, Qiming Zhai, Yong Xia, Luo Suxin

**Affiliations:** ^1^Division of cardiology, The First Affiliated Hospital of Chongqing Medical University, Chongqing 400016, China.; ^2^Department of Cardiothoracic Surgery, The First Affiliated Hospital of Chongqing Medical University, Chongqing 400016, China.; ^3^Institute of Life Science, Chongqing Medical University, Chongqing 400016, China.; ^4^State Key Laboratory of Military Stomatology & National Clinical Research Center for Oral Diseases & Shaanxi International Joint Research Center for Oral Diseases, Center for Tissue Engineering, School of Stomatology, The Fourth Military Medical University, Shaanxi 710032, China.; ^5^Davis Heart and Lung Research Institute, Division of Cardiovascular Medicine, The Ohio State University College of Medicine, OH 43210, USA.

**Keywords:** ketogenic diet, diabetic cardiomyopathy, lifestyle intervention, db/db mice

## Abstract

The ketogenic diet (KD) has been widely used in clinical studies and shown to hace an anti-diabetic effect, but the underlying mechanisms have not been fully elaborated. Our aim was to investigate the effects and the underling mechanisms of the KD on cardiac function in db/db mice. In the present study, db/db mice were subjected to a normal diet (ND) or KD. Fasting blood glucose, cardiac function and morphology, mitochondrial dynamics, oxidative stress, and apoptosis were measured 8 weeks post KD treatment. Compared with the ND, the KD improved glycemic control and protected against diabetic cardiomyopathy in db/db mice, and improved mitochondrial function, as well as reduced oxidative stress were observed in hearts. In addition, KD treatment exerted an anti-apoptotic effect in the heart of db/db mice. Further data showed that the PI3K/Akt pathway was involved in this protective effect. Our data demonstrated that KD treatment ameliorates cardiac dysfunction by inhibiting apoptosis via activating the PI3K-Akt pathway in type 2 diabetic mice, suggesting that the KD is a promising lifestyle intervention to protect against diabetic cardiomyopathy.

Diabetes mellitus (DM) is a metabolic involving the interaction of genetic and environmental factors, and it is characterized by chronic hyperglycemia as the result of impaired insulin secretion, insulin resistance, or both [1, 2]. It has been widely accepted that the increasing prevalence of DM is a serious public health problem. Primarily, cardiovascular diseases, including diabetic cardiomyopathy (DCM), are the leading cause of morbidity and mortality individuals with diabetes [3]. DCM is a serious complication related to DM and is characterized by cardiac remodeling and dysfunction but the absence of coronary atherosclerosis, hypertension and significant vascular disease [4]. Microangiopathy, cardiac fibrosis and disruption of calcium transport are all possible explanations for DCM [5]. In addition, the production of reactive oxygen species and nitrogen species induced directly by hyperglycemia has also been identified as the major promoter of DCM development [6]. Furthermore, inflammation was recently found to play an important role in the pathogenic development of DCM, and anti-inflammatory medications may slow this process [7]. Inflammation and oxidative stress induced by hyperglycemia promote the apoptosis of cardiomyocytes, which leads to myocardial remodeling and subsequent cardiac dysfunction [8]. Many independent pathways may lead to cardiomyocytes apoptosis; some are initiated by ligands that bind to death receptors, while others are governed by the pro-apoptotic proteins released from mitochondria [9]. Phosphatidylinositol 3-kinase (PI3K) exerts an anti-apoptotic effect through activating the Akt pathway, which has been proposed as a potential pathway for the prevention of DCM [10].

Although many drugs are widely used in anti-diabetic treatments, lifestyle interventions such as exercise and caloric restriction have been accepted as a beneficial way to improve the health situation in individuals with diabetes [11]. Caloric restriction induces a shift in metabolism away from carbohydrates toward fat [12]. Interestingly, the ketogenic diet (KD) involves a similar shift in the predominant caloric source predominant caloric source [13]. The KD is a very low-carbohydrate and high-fat diet, and it has been the most widely used dietary intervention for subjects with DM or obesity in clinical studies [14-18].

Although many beneficial effects of the KD had been found in clinical studies, the underlying mechanisms are still poorly understood. Here, we investigated the effects of KD treatment on DCM in db/db mice and found that KD treatment ameliorated cardiac dysfunction by inhibiting apoptosis through activating the PI3K/Akt pathway.

## MATERIAL and METHODS

### Animals

Animals experiments were performed according to the National Institutes of Health Guidelines for Use of Laboratory Animals, and all procedures were approved by Chongqing Medical University Committee on Animal Care. Six-weeks-old male db/db (C57BL/KsJ)and age-matched wil-type mice were purchased from Nanjing Biomedical Research Institute of Nanjing University (Nanjing, China), and housed in temperature-controlled cages (20 °C to 22 °C, fed ad libitum, and maintained on a 12 h light /12 h dark cycle). When they were 8 weeks old, the wild-type and db/db mice were each randomly and equally divided into two groups using a random number table method and then fed on either a control diet or ketogenic diet (see supplementary material Table 1) for 8 weeks [19]. The cardiac and mitochondrial functions were assessed after 8 weeks of dietary treatment. Fasting blood samples were collected to analyze serum glucagon and insulin levels.

### Assessment of cardiac structure and function

Histological studies were performed as described previously [20]. Briefly, cardiac tissues were paraffin embedded and sectioned at a thickness of 5 μm for hematoxylin and eosin staining. Hematoxylin and eosin staining were performed according to the manufacturer’s protocols. Echocardiography to assess cardiac function was performed in the mice using a Vevo 770 High-Resolution Micro-Imaging System (Visual Sonics Inc, Toronto, Canada). Mice were anesthetized with 1% isofluorane/ oxygen administered through inhalation. M-mode and two-dimensional measurements were performed as described previously [21].

### Transmission electron microscopy

Samples were fixed in 2.5% glutaraldehyde in 0.1 M sodium cacodylate buffer (pH 7.2) overnight at 4 °C and processed as described previously [22]. The samples were visualized using a Hitachi microscope (H7500 TEM, Japan). Mitochondrial morphology and number were analyzed using Image-Pro Plus software.

### Respiratory control ratio

A Clark-type oxygen electrode(Strathkelvin 782; Strathkelvin Instruments, UK) was used to evaluate the mitochondrial respiratory control ration by measuring oxygen consumption as described previously [23].Briefly, isolated mitochondria (80-100µg) were mixed with respiration buffer with 2.5 mM succinate added as a substrate, and 2.5 µM rotenone added as a complex I inhibitor. Tge state III and IV respiration rates were measured in the presence or after depletion of 200 μM ADP. The respiratory control ratio was defined as the ratio of the state III to state IV respiratory rate.

### Measurement of ATP, BHB, serum glucagon, insulin and the lipid profile

Cardiac ATP content was measured with a luciferase assay kit (Promega) according to the manufacturer’s instructions and the BHB contents in the heart was measured with β- Hydroxybutyrate Assay Kit kits (Nanjing Jiancheng Reagents, China). Serum glucagon and insulin were measured with a specific commercial kit (ExCell Bio, Shanghai, China). Free fatty acid, triglyceride and total cholesterol levels were measured by using specific commercial kits (Nanjing Jiancheng Reagents, China).

### Determination of malondialdehyde (MDA), manganese superoxide dismutase activity (MnSOD) and caspase-3 activity.

The MDA levels in the myocardium were determined as described previously [24]. MnSOD activities were measured with a determination kit (Beyotime, Shanghai, China) according to the manufacturer’s instructions. Myocardial caspase-3 activity was measured with a caspase cololrimetric assay kits (Chemicon, Temecula, CA, USA) according to the manufacturer’s instruction.

### Cardiomyocytes culture and cell viability determination

Neonatal myocytes were isolated from 1-day-old SD rats and cultured as described previously [25]. Briefly, cells were maintained in DMEM supplemented with 10 % FBS at 37 °C in a humidified incubator with 95 % air and 5 % CO_2_. Cells viability was measured with an MTT cell Cytotoxicity Assay kit (Beyotime, Shanghai, China). The absorbance was read at 570 nm. Six independent experiments were performed.

### Determination of cardiomyocyte apoptosis

Cardiomyocyte apoptosis was determined by terminal deoxynucleotidyl nick-end labeling (TUNEL) assays as described previously [26]. The TUNEL and DAPI double-positive nuclei were counted as apoptotic cells. The apoptotic index was expressed as the number of apoptotic cells / the total number of nucleated cells.

### Detection of superoxide production

The production of superoxide was detected with dihydroethidium (DHE, Sigma, USA) as described previously [27]. The molecules were excited at 488 nm and emission was detected at 610 nm with an inverted confocal microscope (Zeiss LSM 800).

#### Western blotting

Western blotting was used to assess the protein expression and phosphorylation as described previously [28]. The immunoblots were incubated with primary antibodies against anti-PI3K, anti-Akt, anti-p-Akt (Ser473), anti-Bcl2, anti-Bax, anti-Drp1 (Cell Signaling Technology), anti-Mfn1, anti-Mfn2, anti-Opa1 (Abcam), or anti-Fis1 (Genetex) overnight at 4 ? followed by incubation with the corresponding secondary antibodies at room temperature for 1-2 h. The raw bands were detected and quantified with the Bio-Rad imaging system (Bio-Rad, CA, USA).

#### Statistical analysis

All data are presented as the mean ± SEM. The differences in all data were analyzed by two-way ANOVA followed by an unpaired t-test with GraphPad prism version 6.0. P < 0.05 was taken to be statistically significant.

**Table1:** Effects of feeding the KD on the lipid profile and intramyocardial BHB.

	Control	KD	Db	Db+KD
NEFA (µM)	0.60±0.09	0.58±0.02	0.91±0.14*	0.845±0.09
Triglyceride (mg/dL)	84.42±5.16	70.3±3.19	137.65±14.9**	118.28±10.41#
Total cholesterol (mg/dL)	118.28±10.41	132.25±12.83**	172±15.13**	179±10.16
Intramyocardial BHB (mM)	0.5±0.04	2.58±0.37**	0.46±0.04	1.08±0.11#

Values are mean ± SEM; n=4-5 mice. NEFA, nonesterified fatty acid. BHB, beta-hydroxybutyrate.

* , P<0.05.

** , P< 0.01 versus the Control.

# , P<0.05 versus Db.

## RESULTS

### The ketogenic diet improved glycemic control and insulin sensitivity in db/db mice

**Figure 1.**
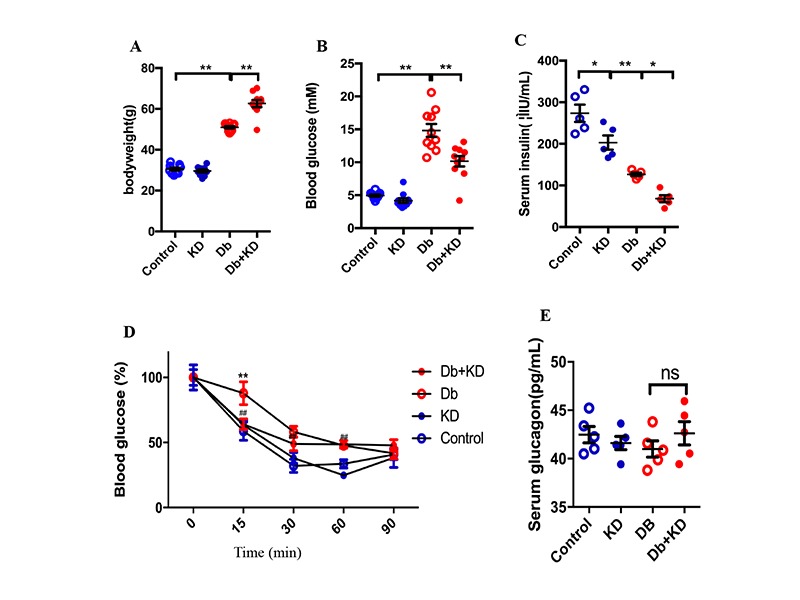
The ketogenic diet improved glycemic control and insulin sensitivity in db/db mice. Body weight (A), fasting blood glucose level (B), serum insulin level (C), insulin sensitivity test (D), and glucagon level (E) of mice after 8 weeks of feeding the KD; n=5-10. Values are the mean ± SEM. Data were analyzed using two-way ANOVA, followed by unpaired t-tests. ^#^, P<0.05. ^##^, P< 0.01 versus Control. *, P<0.05. **, P<0.01 versus Db. ns indicates no significance.

To test whether the KD benefited the management of diabetes mellitus, mice were subjected to a normal diet (Control) or ketogenic diet (KD) for 8 weeks, starting at the age of 8 weeks. Compared with age-matched control mice, db/db mice showed an increased body weight and KD treatment further increased the bodyweight (Figure1 A). These results on body weight were different from those reported in a previous study [29], possibly because of using different diabetic models. Although the body weight increased, feeding the KD did not increased serum NEFA and total cholesterol levels but decreased serum triglyceride levels in db/db mice (Table 1). Interestingly, feeding the KD decreased the levels of fasting blood glucose (Fig. 1B) and serum insulin (Fig. 1C) in db/db mice. Moreover, feeding the KD improved insulin sensitivity as assessed by insulin stimulation tests (Fig. 1D). was not different between the Control and KD groups of diabetic mice (Fig. 1E). These results suggested that the KD benefited glycemic control and improved insulin sensitivity while increasing the degree of obesity in db/db mice.

### The ketogenic diet improved cardiac function and alleviated cardiac remodeling in db/db mice

**Figure 2.**
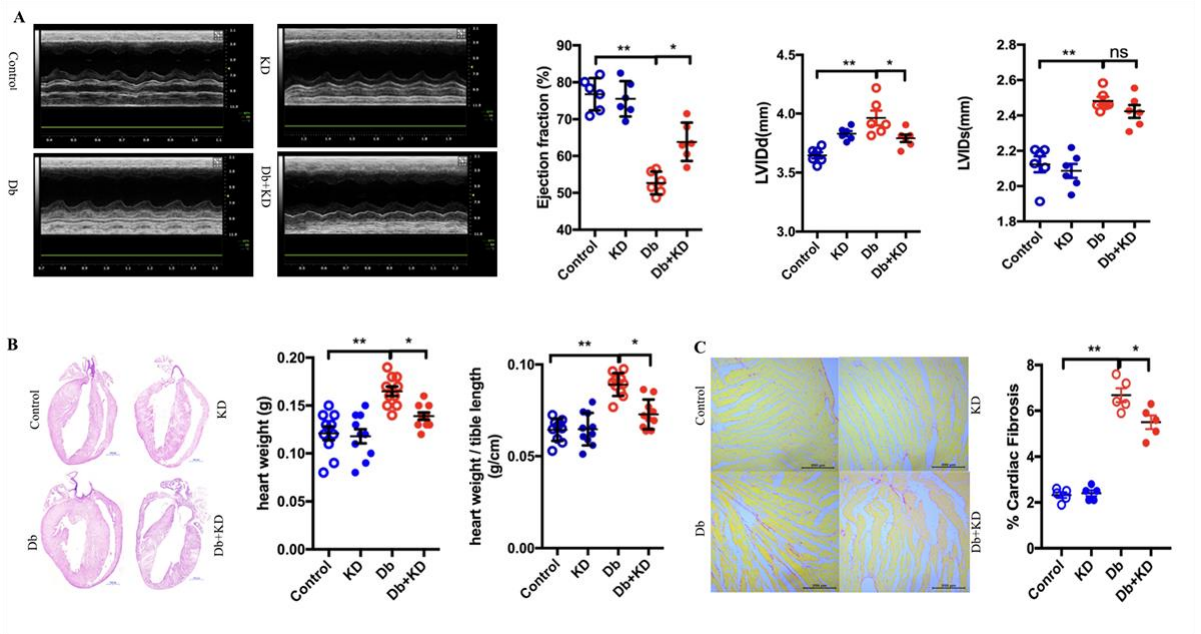
The ketogenic diet improved cardiac function and alleviated cardiac remodeling in db/db mice. A) Feeding the KD improved the cardiac function in db/db mice. Typical echocardiogram images are shown in the left panel. LV ejection fraction, LV internal dimension diastole (LVIDd), and LV internal dimension systole (LVIDs) are shown in the right and lower panels; n=6 mice from each group. B) Cardiac phenotypes in the mice after 2 months of feeding the KD. Four-chamber view cardiac sections stained with hematoxylin and eosin staining. Scale bar, 1 mm. Heart weight and heart weight-to-tibia length ratio are shown in the right panel; n=10 mice from each group. C) Cardiac fibrosis in mice after 2 months of feeding the KD. Sirius red staining. Scale bar, 200μm. Cardiac fibrosis quantification as a percentage of the red stained area vs. total area stained by Sirius red is shown in the right panel; n=5 mice from each group. Values are the mean ± SEM. Data were analyzed using two-way ANOVA, followed by unpaired t-tests. *, P<0.05. **, P<0.01. ns indicates no significance.

To determine whether the improvement in glycemic control provided protection against DCM, cardiac function and morphology were assessed. Compared with the control group, db/db mice exhibited impaired cardiac function as evidenced by a decreased left ventricular rejection fraction (LVEF) and an increased left ventricular internal dimension (LVID) (Fig. 2A). In addition, db/db mice had an increased heart weight and heart weight-to-tibia length ratio (Fig. 2B). Feeding the KD increased LVEF and normalized LVID in db/db mice (Fig. 2A) and also aignificantly decreased the heart weight and weight-to-tibia length ratio (Fig. 2B). Fibrosis is a major feature of diabetic cardiomyopathy, and our data demonstrated that feeding the KD reduced the level of cardiac fibrosis in db/db mice (Fig. 2C). These findings indicated that the KD exerted cardioprotective effects in db/db mice.

### The ketogenic diet prevented mitochondrial fission and improved mitochondrial function in the myocardium of db/db mice

**Figure 3.**
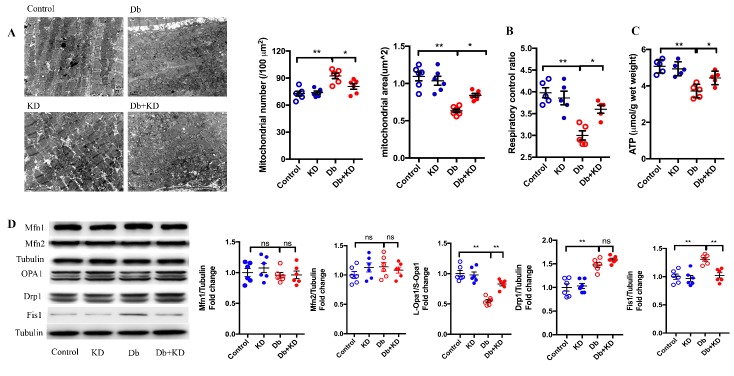
The ketogenic diet prevented mitochondrial fission and improved mitochondrial function in the myocardium of db/db mice. A) Cardiac mitochondrial morphology in myocardial samples. Typical images obtained from electron microscopy are shown in the left panel. Scale bar, 1 μm. Average mitochondrial size and mitochondrial number are shown in the right panel; n=6 mice from each group. B) Mitochondrial respiratory control ration; n=5 mice from each group. C) ATP levels in the heart of the mice; n=5 mice from each group. D) The content of mitochondrial dynamics-related proteins in myocardial samples. Statistical results are shown in the right panel; n=6 mice from each group. Values are the mean ± SEM. Data are analyzed using two-way ANOVA, followed by unpaired t-tests. *, P<0.05. **, P<0.01. ns indicates no significance.

It has been suggested that mitochondrial dynamics plays a pivotal role in cardiac function [30]. We next determined whether the KD regulated mitochondrial morphology and function in the heart of db/db mice. As expected, diabetes resulted in cardiac mitochondrial fragmentation as evidenced by increased mitochondrial number and decreased mitochondrial size in db/db mice (Fig. 3A). The mitochondrial respiratory control ratio (Fig. 3B) and ATP content (Fig. 3C) were also significantly decreased in db/db mice. Notably, feeding the KD prevented mitochondrial fission and improved mitochondrial function in db/db mice (Fig. 3A-C). These findings suggested that feeding the KD rebalanced mitochondrial dynamics and function in db/db mice.

To investigate the underlying mechanism of these effects of the KD on mitochondrial dynamics by KD, we measured the levels of the main mitochondrial dynamics-related proteins (Fig. 3D). There was no difference in the content of Mfn1 and Mfn2. It has been proven that L-Opa1 contributes to mitochondrial fusion and that S-Opa1 is required for mitochondrial fission. The ratio of L-Opa1/S-Opa1 was decreased in the heart of db/db mice, and the KD significantly increased this ratio. Both Drp1 and Fis1 promote mitochondrial fission, and the contents of Drp1 and Fis1 increased in db/db mice. Feeding the KD decreased the content of Fis1 in db/db mice but did not affect the levels of Drp1. These results indicated that feeding the KD inhibited mitochondrial fission and improved mitochondrial function via regulating the expression levels of mitochondrial dynamics-related proteins.

### The ketogenic diet suppressed oxidative stress in the heart of db/db mice

**Figure 4.**
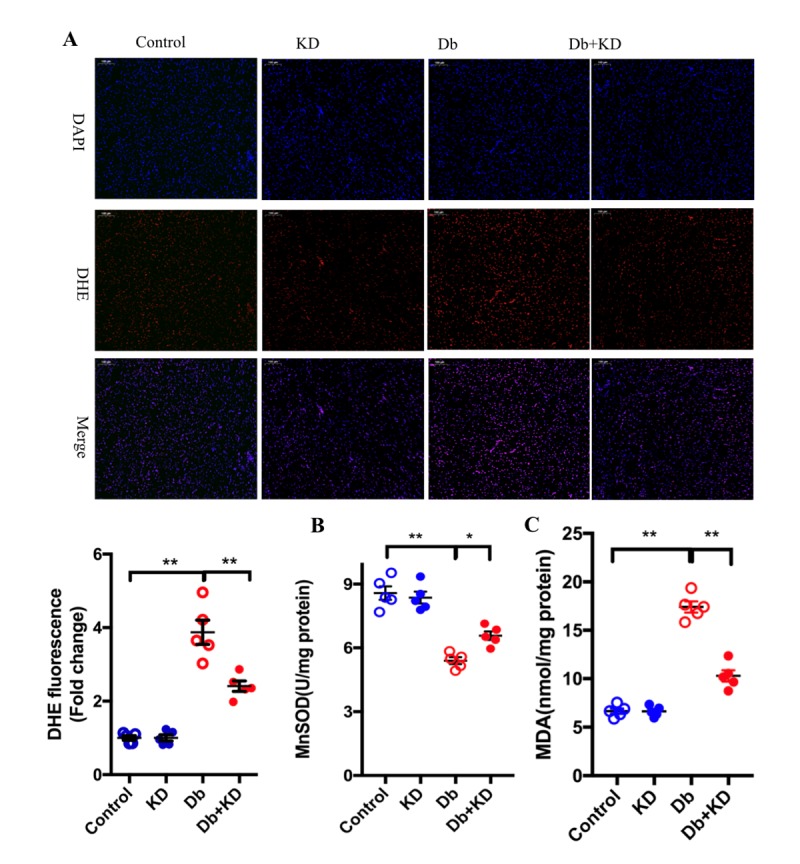
The ketogenic diet suppressed oxidative stress in the heart of db/db mice. A) Representative fluorescence microscopic image of DHE staining (red fluorescence) and DAPI-staining (blue fluorescence). Results derived from 5 different mice are shown below. Scale bar, 100 μm. Feeding the KD increased the activity of MnSOD (B) and decreased the content of MAD (C) in the myocardium of db/db mice; n=6 mice from each group. Values are the mean ± SEM. Data were analyzed using two-way ANOVA, followed by unpaired t-tests. *, P<0.05. **, P<0.01 versus Control.

ROS are generated during the process of mitochondrial respiration when electrons are leaked to O_2_ at some sites in the electron transport chain [31]. Unbalanced mitochondrial dynamics is one factor that leads to ROS overproduction. Thus, we examined the levels of ROS. Indeed, the myocardium of db/db mice showed a marked increase in ROS generation as evidenced by DHE staining (Fig. 4A). MDA is a major product of lipid peroxidation and MnSOD is a main antioxidant enzyme that protects against oxidative damage. Both MDA and MnSOD can serve as indicators of oxidative stress. Indeed, a decreased MnSOD level and increased MDA production were found in the heart of db/db mice (Fig. 4B and C). In contrast, feeding the KD decreased ROS and MDA generation but partly increased MnSOD activity partly. These results indicated that the KD suppressed oxidative stress in the heart of db/db mice.

### The ketogenic diet attenuated cardiocyte apoptosis in db/db mice

**Figure 5.**
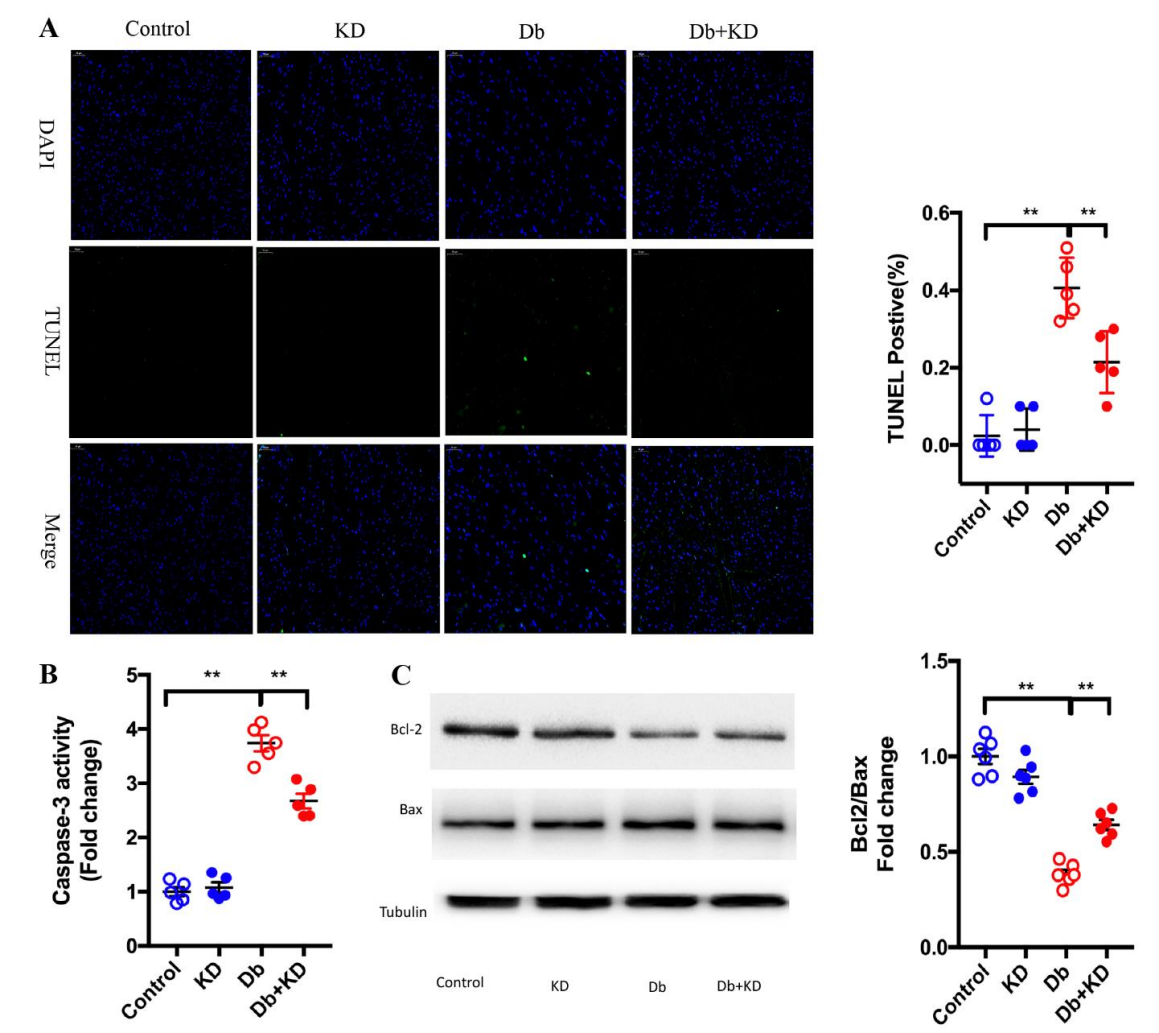
The ketogenic diet attenuated cardiocyte apoptosis in db/db mice. A) Top representative TUNEL-stained (green fluorescence) and DAPI-stained (blue fluorescence) photomicrographs. Scale bar, 50 μm. Results derived from 5 different mice are shown in the right panel. B) Feeding the KD decreased caspase-3 activity. C) Feeding the KD increased the ratio of Bcl-2/Bax in the myocardium of db/db mice; n=6 mice from each group. Values are the mean ± SEM. Data were analyzed using two-way ANOVA, followed by unpaired t-tests. *, P<0.05. **, P<0.01.

Mitochondrial dynamics plays a central role in the maintenance of the cell proliferation-apoptosis balance [32]. We next determined the effects of the KD on cardiomyocyte apoptosis under diabetic conditions. We found that compared to control mice, db/db mice had a significantly increased apoptosis level (Fig. 5A) and caspase-3 activity (Fig. 5B). Additionally, the ratio of Bcl-2/Bax was decreased in myocardium samples from diabetic hearts (Fig. 5C). Treatment with the KD significantly decreased the apoptosis index and caspased-3 activity but increased the ratio of Bcl-2/Bax. These results indicated that KD treatment attenuated cardiocyte apoptosis in ab/db mice, which may contribute the improved cardiac function and remodeling described above.

### BHB attenuated high-glucose-induced apoptosis in cardiomyocytes through the PI3K/Akt pathway

**Figure 6.**
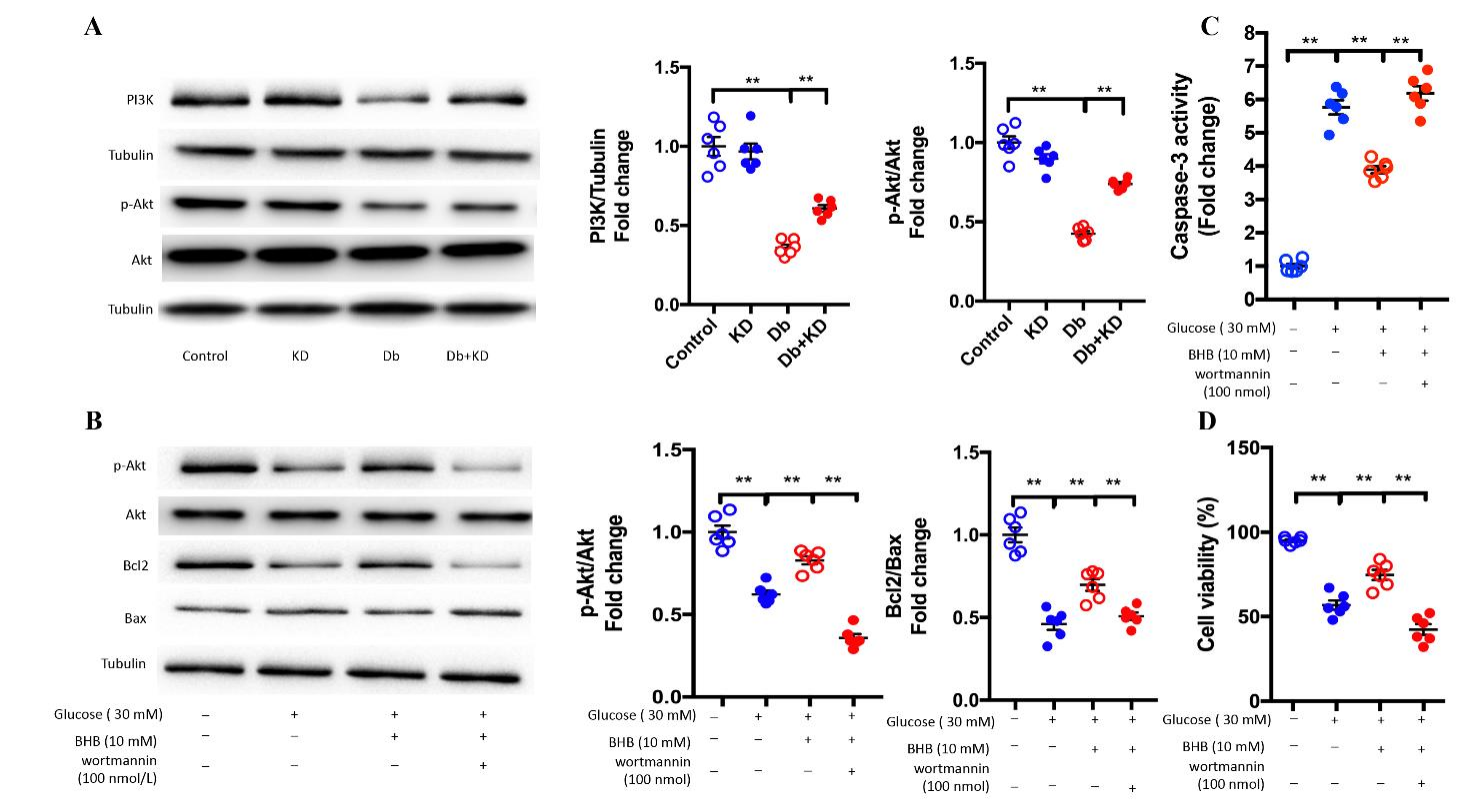
BHB attenuated high glucose induced apoptosis in cardiomyocytes through the PI3K/Akt pathway. A) The KD increased the cardiac contents of PI3K and the ration of p-Akt/Akt in the myocardium of db/db mice. The typical images of western blot images are shown in the left panel. The statistical results are shown in the right panel; n=6 mice from each group. B) Effects of BHB on increasing the ration of Bcl2/Bax and p-Akt/Akt were blunted by the PI3K/Akt inhibitor wortmannin in cardiomyocytes exposed to high glucose; n=6. C) BHB decreased caspase-3 activity in cardiomyocytes exposed to high glucose; n=6. D) BHB protected cardiomyocytes against high glucose-induced apoptosis; n=6. Values are the mean ± SEM. Data were analyzed using two-way ANOVA, followed by unpaired t-tests. *, P<0.05. **, P<0.01.

It has been demonstrated that the PI3K/Akt pathway plays a crucial role in the prevention of apoptosis [33]. To investigate the underlying mechanisms of the anti- apoptotic effects of the KD, we measured the contents of PI3K, p-Akt and Akt in myocardium samples. The content of PI3K and the ratio of p-Akt/Akt were markedly decreased in db/db mice, and these changes were restored partly by KD treatment (Fig. 6A). Many studies have demonstrated that KD treatment enhances the levels of the ketone body metabolite beta-hydroxybutyrate (BHB) [34], and our data showed the KD increased the myocardium BHB level (Table 1) ; Thus, we next performed studies to determine whether this ketone body had direct anti- apoptotic effects in neonatal rat cardiomyocytes exposed to high glucose (30 mM) for 10-12 hours.. It was found that high glucose resulted in a significant decrease in the ratio of p-Akt/Akt, accompanied by a decrease in the ratio of Bcl2/Bax and an increase in caspase-3 activity (Fig. 6B and C), while treatment with the ketone body BHB (10 mM) treatment not only increased the ratio of p-Akt/Akt and Bcl2/Bax but also decreased the activity of caspases-3 in isolated cardiomyocytes. More importantly, BHB improved cell viability in neonatal rat cardiomyocytes exposed to high glucose for 24 hours (Fig. 6D). All these protective effects were blunted and even reversed by the PI3K inhibitor wortmannin (100 nM) (Fig. 6A-D), suggesting that BHB exerted protective effects via the PI3K-Akt pathway. Those results indicated that the KD protects the hearts from DCM partly through the PI3K-Akt pathway.

## DISCUSSION

Endocrine and metabolic disruptions play a significant role in the initiation and progression of DM. It has been proposed that dietary intervention should be a primary strategy in the clinical management of DM [35]. In this study, we found that KD treatment ameliorated cardiac dysfunction and remodeling in db/db mice. The ketone body BHB was found to have an anti-apoptotic effect on the diabetic myocardium via the PI3K/Akt pathway. These findings, together with those indicating that feeding mice the KD preserved mitochondrial function and decreased oxidative stress, suggesting the potential for the use of ketone bodies in anti-diabetic therapy.

Various studies have demonstrated that the KD shifts the metabolism substrate from carbohydrates to fatty acids in individuals with diabetes and benefits the management of DM clinically [36]. Some studies on animals have also shown that the KD can prevents the development of diabetes [37, 38].In addition, a recent animal study showed that the KD extends longevity and health span in adult mice [13]. Another study found that the KD reduces midlife mortality and improves memory in aging mice [19]. Our previous work demonstrated that feeding a high-fat diet (45% of calories derived from fatty acids) feeding for 2 months after transverse aortic constriction (TAC) surgery enhanced fatty acid utilization and prevented the development of heart failure in mice [20]. These findings suggested that the KD, a high-fat and low-carbohydrate diet, may exert health-promoting effects in both humans and animals.

In this study, we observed that feeding mice the KD decreased blood glucose and improved insulin sensitivity. However, we observed that feeding the KD did not reduce but increased body weight in diabetic mice, which is different from the reported in a previous study [29]. The difference in the diabetic models used may this difference, as another study performed on ob/ob mice also showed that feeding the KD increased bodyweight as well [39]. Regardless, our data suggested that feeding the KD ameliorates cardiac dysfunction and remodeling in db/db mice.

It has been demonstrated that mitochondria play an important role in DCM [40]. Mitochondrial dynamics, which are regulated by several specific mitochondrial fission and fusion proteins, determine mitochondrial morphology and function [41]. It has been reported that there are links between mitochondrial dynamics and feeding the KD [42]. In this diabetic model, the significant cardiac dysfunction and myocardial remodeling were accompanied by mitochondrial fission and dysfunction. In accordance with this, our results indicated that mitochondrial became fragmented and that their function was impaired, as evidenced by decreased ATP content and respiration control rate. Feeding the KD inhibited mitochondrial fission and improved mitochondrial function, which may be an underlying mechanism contributing to the protection of cardiac function. Mfn1/2 and Opa1(mediate mitochondrial fusion), as well as Drp1 and Fis1 (mediate mitochondrial fission) are abundant in cardiac tissue [43]. We found that feeding the KD significantly increased the ratio of L-Opa1/S-OPa1 and decreased the levels of Fis1, indicating that these two proteins may be promising targets for further studies on the prevention of DCM.

In addition to supplying energy, mitochondria have been recognized as a main source of ROS in the myocardium. Mitochondrial dysfunction results in excessive ROS generation [44]. In this study, we found increased ROS generation in the myocardium accompanied by increased MDA production and decreased MnSOD activity in diabetic mice. Feeding the KD suppressed the ROS production and increased MnSOD activity and reduced the MDA level in the heart of db/db mice, suggesting that the KD exerted an anti-oxidative stress effects in diabetic mice. However, whether this is a direct effect or a benefit from improved mitochondrial function requires further study.

Mitochondrial dynamics are involved in many cellular processes, including apoptosis, which plays a crucial role in the process of DCM [45, 46]. Mitochondrial fragmentation is related to many forms of cell death, but mitochondrial fusion protects cell from apoptosis. The PI3K/Akt pathway plays important roles in preventing apoptosis, and inhibition of this pathway contributes to the development of DCM [47]. In the present study, the content of PI3K and the ratio of p-Akt/Akt were significantly lower but apoptosis was active in myocardium samples from db/db mice compared with those in samples from control mice. KD treatment not only markedly reversed the changes in the content of PI3K and the ratio of p-Akt/Akt but also suppressed myocardial apoptosis as evidenced by the TUNEL results. Additionally, in isolated cardiomyocytes, we confirmed that the ketone body BHB activated the PI3K/Akt pathway, thus increasing the ration of Bcl2/Bax and decreasing the activity of caspase-3. Wortmannin, a PI3K inhibitor, eliminated the anti-apoptotic effects of BHB in cardiomyocytes, suggesting that the KD exerted anti-apoptotic effects via activating the PI3K-Akt pathway in diabetic mice.

However, our study had several limitations. We investigated the effects of the KD in db/db mice, but the efficacy of the KD in other diabetic models still needs to be elucidated in future studies. In addition, we clarified the anti-apoptosis mechanism in vitro using an inhibitor of PI3K, but studies performed with Akt conditional knockout mice would generate more direct evidences. Furthermore, the specific mechanism by which the KD regulates glucose homeostasis should be further explored.

### Conclusions

Taken together, the KD not only balanced glucose homeostasis but also alleviated cardiac dysfunction by inhibiting apoptosis via the activating PI3K-Akt pathway in type 2 diabetic mice. Therefore, the KD may be a promising lifestyle intervention for the management of diabetes mellitus to retard the progression of diabetic cardiomyopathy.

## Supplementary Materials

The Supplemenantry data can be found online at: www.aginganddisease.org/EN/10.14336/AD.2019.0510.
