# Supplementary material for: Ketogenic Diet Ameliorates Cardiac Dysfunction via Balancing Mitochondrial Dynamics and Inhibiting Apoptosis in Type 2 Diabetic Mice
Source: Aging Dis. 2020 Mar 9;11(2):229–40. doi: 10.14336/AD.2019.0510 (PMC7069456; doi:10.14336/AD.2019.0510)
Supplement: Supplementary file 1 — The Supplemenantry data can be found online at: www.aginganddisease.org/EN/10.14336/AD.2019.0510. [file AD-11-2-229-s.pdf]

SUPPLEMENTARY DATA

Ketogenic Diet Ameliorates Cardiac Dysfunction via Balancing Mitochondrial Dynamics and Inhibiting Apoptosis in Type 2 Diabetic Mice

Yongzheng Guo[1,#], Cheng Zhang[2,#], Feifei Shang[3], Minghao Luo[1], Yuehua You[1], Qiming Zhai[4], Yong Xia[1,3,5], Suxin Luo[1,*]

[1]Division of cardiology, The First Affiliated Hospital of Chongqing Medical University, Chongqing 400016, China. [2]Department of Cardiothoracic Surgery, The First Affiliated Hospital of Chongqing Medical University, Chongqing 400016, China. [3]Institute of Life Science, Chongqing Medical University, Chongqing 400016, China. [4]State Key Laboratory of Military Stomatology & National Clinical Research Center for Oral Diseases & Shaanxi International Joint Research Center for Oral Diseases, Center for Tissue Engineering, School of Stomatology, The Fourth Military Medical University, Shaanxi 710032, China. [5]Davis Heart and Lung Research Institute, Division of Cardiovascular Medicine, The Ohio State University College of Medicine, OH 43210, USA.

© 2019. Guo Y et al. Published online at http://www.aginganddisease.org/EN/10.14336/AD.2019.0510

SUPPLEMENTARY DATA

Supplementary Table 1. Compositions of experimental diets

Ingredient (g/kg diet)	Control diet	Ketogenic diet
Casein	100	180
DL-methionine	1.6	2.88
Corn starch	512.46	0
Sucrose	100	0
Maltodextrin	155	0
Crisco	25	440
Cocoa butter	0	150
Corn oil	25	85
Cellulose	35	9.19
Calories per gram	3.7	6.7

Supplementary Table 2. Echocardiography result of mice after ND or KD feeding.

	Control	KD	Db	Db+KD
Heart rate	475.8±10.6	479.2±9.9	481.3±6.7	470.2±11.1
LVIDd	3.64±0.06	3.83±0.05	3.96±0.14**	3.79±0.12#
LVIDS	2.10±0.11	2.09±0.09	2.52±0.06**	2.35±0.08
LVPWd	0.62±0.05	0.66±0.04	0.73±0.03**	0.66±0.03#
LVPWs	1.04±0.12	1.02±0.11	1.18±0.10	1.15±0.07
IVSd	0.75±0.05	0.74±0.12	0.79±0.13	0.76±0.07
IVSs	0.96±0.21	1.02±0.18	1.14±0.05	1.07±0.13
LV mass	94.25±8.94	93.42±8.48	118.5±8.23*	105.73±23.11

© 2019. Guo Y et al. Published online at http://www.aginganddisease.org/EN/10.14336/AD.2019.0510
